# Physical mapping resources for large plant genomes: radiation hybrids for wheat D-genome progenitor *Aegilops tauschii*

**DOI:** 10.1186/1471-2164-13-597

**Published:** 2012-11-05

**Authors:** Ajay Kumar, Kristin Simons, Muhammad J Iqbal, Monika Michalak de Jiménez, Filippo M Bassi, Farhad Ghavami, Omar Al-Azzam, Thomas Drader, Yi Wang, Ming-Cheng Luo, Yong Q Gu, Anne Denton, Gerard R Lazo, Steven S Xu, Jan Dvorak, Penny MA Kianian, Shahryar F Kianian

**Affiliations:** 1 Department of Plant Sciences, North Dakota State University, Fargo, ND, 58108, USA; 2 Department of Computer Sciences, North Dakota State University, Fargo, ND, 58105, USA; 3 USDA-ARS, Western Regional Research Center, Albany, CA, 94710, USA; 4 Department of Plant Sciences, University of California, Davis, CA, 95616, USA; 5 USDA-ARS, Northern Crop Science Laboratory, Fargo, ND, 58102, USA; 6 Present address: Math, Science and Technology Department, University of Minnesota, Crookston, MN, 56716, USA

**Keywords:** *Aegilops tauschii*, Genetically effective cell number, Physical mapping, Radiation hybrid mapping, Repeat DNA junction marker, Wheat

## Abstract

**Background:**

Development of a high quality reference sequence is a daunting task in crops like wheat with large (~17Gb), highly repetitive (>80%) and polyploid genome. To achieve complete sequence assembly of such genomes, development of a high quality physical map is a necessary first step. However, due to the lack of recombination in certain regions of the chromosomes, genetic mapping, which uses recombination frequency to map marker loci, alone is not sufficient to develop high quality marker scaffolds for a sequence ready physical map. Radiation hybrid (RH) mapping, which uses radiation induced chromosomal breaks, has proven to be a successful approach for developing marker scaffolds for sequence assembly in animal systems. Here, the development and characterization of a RH panel for the mapping of D-genome of wheat progenitor *Aegilops tauschii* is reported*.*

**Results:**

Radiation dosages of 350 and 450 Gy were optimized for seed irradiation of a synthetic hexaploid (AABBDD) wheat with the D-genome of *Ae. tauschii* accession AL8/78. The surviving plants after irradiation were crossed to durum wheat (AABB), to produce pentaploid RH_1_s (AABBD), which allows the simultaneous mapping of the whole D-genome. A panel of 1,510 RH_1_ plants was obtained, of which 592 plants were generated from the mature RH_1_ seeds, and 918 plants were rescued through embryo culture due to poor germination (<3%) of mature RH_1_ seeds. This panel showed a homogenous marker loss (2.1%) after screening with SSR markers uniformly covering all the D-genome chromosomes. Different marker systems mostly detected different lines with deletions. Using markers covering known distances, the mapping resolution of this RH panel was estimated to be <140kb. Analysis of only 16 RH lines carrying deletions on chromosome 2D resulted in a physical map with *cM/cR* ratio of 1:5.2 and 15 distinct bins. Additionally, with this small set of lines, almost all the tested ESTs could be mapped. A set of 399 most informative RH lines with an average deletion frequency of ~10% were identified for developing high density marker scaffolds of the D-genome.

**Conclusions:**

The RH panel reported here is the first developed for any wild ancestor of a major cultivated plant species. The results provided insight into various aspects of RH mapping in plants, including the genetically effective cell number for wheat (for the first time) and the potential implementation of this technique in other plant species. This RH panel will be an invaluable resource for mapping gene based markers, developing a complete marker scaffold for the whole genome sequence assembly, fine mapping of markers and functional characterization of genes and gene networks present on the D-genome.

## Background

Wheat is one of the major food crops grown worldwide. Its genetic improvement holds the key to achieving the levels of production necessary to meet the demands of an ever increasing world population. Keeping this in mind, the International Wheat Genome Sequencing Consortium (IWGSC; http://www.wheatgenome.org/) was established with the goal to fully sequence the wheat genome. Once achieved, the genome sequence of wheat will empower plant biologists and plant breeders worldwide to better understand the biology underlying important traits and consequently, their improvement using modern molecular biology tools.

Bread wheat (*Triticum aestivum* L.), which accounts for ~95% of world wheat production, is evolutionarily the youngest polyploid (segmental allohexaploid) species among the cultivated crops. It has a large genome (~17 Gb) which is about eight times larger than that of maize (*Zea mays* L.) and 40 times that of rice (*Oryza sativa* L.) [1]. The large genome size and presence of over 80% repetitive sequences [2] makes the development of a complete physical map, a necessary first step to whole genome assembly of this species, a formidable challenge.

To partially overcome the difficulties associated with the assembly of the wheat genome, the IWGSC has approached sequencing through the construction of BAC based physical maps of individual chromosomes. Once physical contigs of acceptable size (~ 0.5-1 Mb) are created, markers will be developed from each contig and used to anchor them to a genetic marker scaffold. However, it has been shown that the distribution of crossing over events along the length of wheat chromosomes is uneven; very low in the centromeric regions and generally increases towards the telomeric regions [3-7]. By some estimates one-third of the wheat genome, present around the centromeres, accounts for less than 1% of the total recombination [5]. Additionally, large segmental blocks with very low recombination rates are scattered throughout the genome leading to difficulties in estimating accurate genetic to physical map distances. Thus, recombination-based mapping will not provide the necessary coverage and resolution to anchor a physical map and to estimate gap sizes between contigs. Cytogenetic stocks in wheat (e.g., ditelosomic lines and deletion bin lines) have been used for mapping and anchoring of BAC contigs [8]. However, these stocks provide limited resolution due to a large deletion size (average deletion bin is ~35 Mb) and the inability to order markers within a given bin [8]. Radiation hybrid (RH) mapping is a valuable alternative to recombination-based maps and has been adopted for scaffold assembly of numerous animal genomes [9].

Radiation hybrid mapping, originally developed for mapping the human genome [10], employs radiation-induced chromosomal breaks rather than genetic recombination to map markers onto chromosomes. In principle, the likelihood of the radiation-induced DNA breaks to separate two adjacent markers increases with the increase in physical distance between the two. Therefore, the estimation of frequency of deletions/retention between markers as well as the deletion/co-retention patterns of various markers determines the distance between them and their order. Radiation is expected to effect the genome randomly, independent of chromosomal location; thus making the RH mapping a powerful tool for ordering markers and genes in any chromosomal region [11]. Additionally, the marker scoring in RH mapping is based on presence (retention) *vs.* absence (deletion) and does not require allelic polymorphism. This facilitates mapping of gene-based markers, such as ESTs, which are commonly low in polymorphism [12] and are valuable tools for interspecies comparative studies. Moreover, a very high mapping resolution (< 200 Kb) can be achieved using relatively small number of RH lines [13-16].

Radiation hybrid mapping, in combination with recombination mapping, has contributed enormously towards whole genome physical analysis and sequence assembly of human and animal genomes [9,17]. However, there have been relatively few examples of using RH mapping in plants, which include maize [18,19], barley (*Hordeum vulgare* L.; [20]), cotton (*Gossipium hirsutum* L.; [21,22]), and wheat [8,14,23-25]. Despite limited reports, RH mapping in plants has shown the ability to uniquely map markers that could not be resolved through traditional genetic mapping [21], and has potential to order BAC contigs into complete physical maps [25].

The D-genome, which is the smallest among the three genomes of bread wheat, is known to harbor genes/QTL for several important traits including yield [26], resistance to diseases [27], growth [28] and domestication [29]. The International Triticeae Mapping Initiative (ITMI) population was developed using W7984, a synthetic wheat derived from a cross between Altar 84 × *Aegilops tauschii*[30], and has been used extensively to study numerous quantitative traits [26,29,31-34]. Studies on this population showed that the D-genome of *Ae. tauschii* harbors positive alleles for many important traits. However, since the D-genome is a recent evolutionary addition to the hexaploid wheat genome (> 10,000 years old), there has been limited gene flow from *Ae. tauschii* to cultivated wheat [35]. Due to this fact, wheat varieties show limited marker polymorphism among D-genome loci [12]. Thus, attempts to develop saturated D-genome genetic maps would inevitably encounter the strenuous problem of limited polymorphic markers. Such factors laid the foundations for initiatives to develop physical maps of the diploid ancestral progenitor of the D-genome, in the hope that physical maps of *Ae. tauschii* would simplify development of physical maps of bread wheat. The accession ‘AL8/78’ of *Ae. tauschii* was selected as it has been reported to have high genetic similarity to the D-genome of bread wheat [36]. It was used to develop numerous mapping resources including BAC libraries [37], high density gene-based genetic maps [38] and about one half million SNPs [39]. Efforts are currently underway to develop a physical map of this accession (http://wheat.pw.usda.gov/PhysicalMapping, http://www.wheatgenome.org/). However, due to non-homogenous recombination along the length of the chromosomes and the low level of polymorphism for the D-genome in the cultivated pool, genetic mapping alone is not likely to result in a saturated marker scaffold. Therefore, in the present study, a RH panel was developed for the *Ae. tauschii* accession AL8/78 in order to complement available data from genetic mapping and potentially produce a complete physical map of the D-genome. Here, the development of this panel (referred to as AL8/78-DGRH_1_ panel) of >1,500 lines and its detailed molecular characterization is reported. The utility of this panel in mapping gene-based markers and BAC contigs is demonstrated and the importance of this new resource in the functional analysis of important genes is discussed.

## Results

### Effective radiation dose and AL8/78-DGRH_1_ panel development

**Figure 1:**
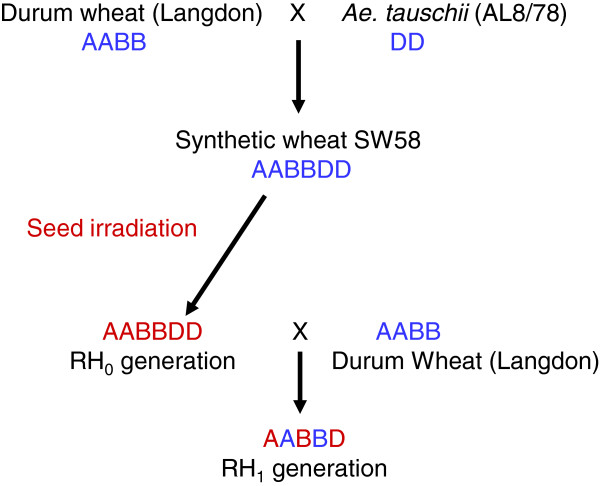
**Schematic presentation of RH**_**1**_** panel development for the D-genome of *Ae. tauschii* accession AL8/78.**

**Figure 2:**
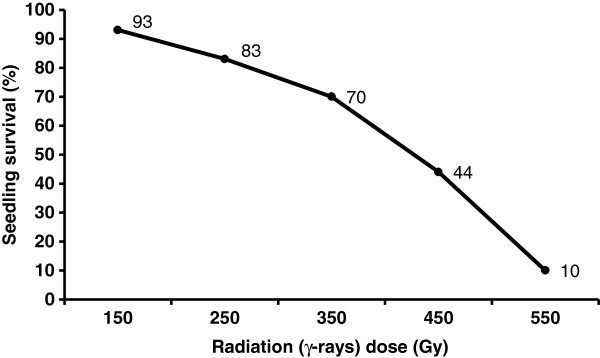
**Effect of γ-rays on seedling survival of synthetic hexaploid wheat.** Batches of 100 seeds were irradiated at various dosages and plant survival for each dose was determined as a proportion of surviving seedlings (%) compared to the survival of plants from control seeds after 1 month.

In order to develop a RH panel for the D-genome of *Ae. tauschii* accession AL8/78, seeds of a synthetic hexaploid wheat 'SW58' (*Triticum aestivum* L.; 2n=42; AABBDD) produced by crossing durum wheat cultivar ‘Langdon’ (LDN; *Triticum durum* L*.*; 2n=28; AABB) to AL8/78 (*T. tauschii* L; 2n=14; DD) and doubling the resulting haploid progeny [40], were γ-irradiated. The resulting RH_0_ plants were crossed back to LDN to generate the RH_1_ panel for the D-genome (DGRH_1_; Figure 1). The first step in RH panel (or any mutation population) development is the determination of optimum dose for irradiation as it may vary based on the target tissue and the genotype. In plant RH systems, the optimum dosage is the amount of irradiation that assures sufficient deletions without causing damage so serious that cannot be tolerated by the organism either during development or at the time of reproduction. To find the effective dose for the synthetic wheat SW58, batches of 100 seeds were irradiated at five different levels of γ-rays (Figure 2). Higher survival rates were observed at low doses of irradiation {150 and 250 Gray (Gy)}, and survival rates decreased sharply at higher doses (450 and 550 Gy; Figure 2). The survival rate at 150, 250, 350, 450, and 550 Gy was 93, 83, 70, 44 and 10%, respectively. Therefore, doses of 350 Gy and 450 Gy were selected as optimal, and used to irradiate more seeds for development of the DGRH_1_ panel.

Initially, attempts were made to develop the population by crossing irradiated SW58 as male with LDN as female, for practical reasons related to emasculation. The planting of ~5,000 RH_1_ seeds belonging to 392 LDN (♀) × SW58 (♂) crosses showed very low germination (~3%). Therefore, in the next set of crosses, irradiated SW58 was used as female and LDN as male. Germination of the RH_1_ seeds increased to ~35%. To further improve the germination, mature embryos were rescued using tissue culture. Embryo rescue resulted in recovery of >80% developing seeds in case of SW58 (♀) × LDN (♂) crosses. However, no improvement was observed using embryo rescue in case of LDN (♀) ×SW58 (♂) crosses.

A total of 1,510 DGRH_1_ plants were obtained, of which 161 belonged to LDN × SW58 cross, whereas 1,349 RH_1_ belonged to SW58 × LDN cross. A total of 592 RH_1_ plants were generated from the mature RH_1_ seeds, and 918 RH_1_ plants were obtained through embryo culture.

### Characterization of the AL8/78-DGRH_1_ panel

#### Characterization using 35 whole genome SSR markers

To determine the extent to which the D-genome chromosomal fragments were deleted in the AL8/78-DGRH_1_ panel, the whole panel was characterized using 35 SSR markers selected from across the seven D-genome chromosomes. These SSR markers represent different deletion bins of each chromosome ensuring even distribution across the genome [41,42]. An average of 2.1% marker loss was observed using this set of 35 markers. An increase in average marker loss (from 1.2 to 2.4%) was observed with an increase in radiation doses from 150 to 450 Gy (Table 1). The average marker loss for individual chromosome in this panel ranged from 2.03% (7D) to 3.01% (4D) and was found to be homogenous by *χ*^2^ homogeneity test. The frequency of individual marker loss ranged from 0.89-3.95% and was heterogeneous across the genome (*p*<0.01). However, the *χ*^2^ homogeneity test suggested that the frequency of individual marker loss in a given chromosome was homogeneous for all D-genome chromosomes except for chromosomes 1D and 7D. In case of chromosome 1D, two markers, cfd19 (present in the deletion bin 1DL2-0.41-1.00) and cfd21 (present in the deletion bin 1DS5-0.70-1.00) showed significantly lower marker loss (0.89 and 1.42% respectively) than others (3.15-3.95%). For chromosome 7D, heterogeneity was due to marker barc1046 which showed higher marker loss (3.02%) than other four markers (1.48-1.87%).

**Table 1:** **Characterization of the AL8/78-DGRH**_**1 **_**panel**

**Dosage (Gy)**	**DGRH**_**1 **_**lines**	**Frequency of marker loss (%)**	Frequency of lines with deletions (%)^*^
150	139	1.2	14.8
250	82	1.7	24.4
350	676	1.9	24.5
450	613	2.4	25.9

^*^Data based on screening of the panel with 35 SSR markers. No lines survived 550 Gy irradiation dose.

In this AL8/78-DGRH_1_ panel, whole genome marker loss for individual RH_1_ lines ranged from 0–42.9%, while the marker loss for individual chromosomes ranged from 0-100%. There were no significant differences in the proportion of individuals with deletions among the 250, 350 and 450 Gy lines, although a slight increase was observed with the increase in dosage (Table 1). A significantly (*p*<0.05) lower number of individuals with deletions (14.8%) were observed for 150 Gy lines compared to 250, 350 and 450 Gy lines. Chi-square homogeneity test showed that the proportion of lines with deletions for individual chromosome, which ranged from 4.0% (3D) to 7.50% (1D), was heterogeneous (*p*<0.001) (Table 2).

**Table 2:** **Marker loss and lines with deletions for individual D-genome chromosomes in the AL8/78-DGRH**_**1 **_**panel**

**Chromosome**	**Frequency of marker loss% (Range)**	**Lines with deletions (%)**
1D	2.60 (0.90-3.95)	7.50
2D	2.84 (2.07-3.20)	4.90
3D	2.31 (2.10-2.58)	4.00
4D	3.01 (2.45-3.20)	4.10
5D	2.65 (2.37-3.12)	4.35
6D	2.88 (2.22-3.20)	5.80
7D	2.03 (1.48-3.02)	4.35

**Figure 3:**
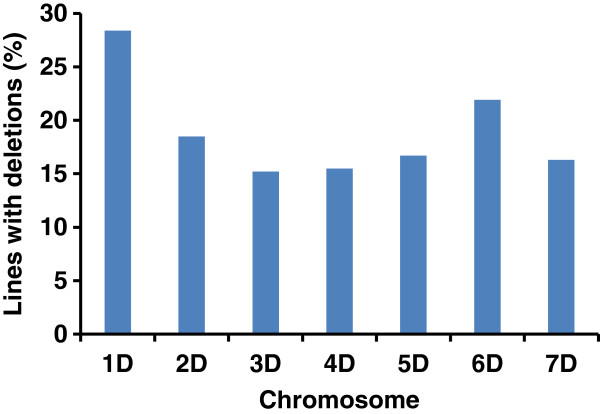
Distribution of informative RH lines for individual chromosomes in the selected RH panel.

Three hundred ninety-nine lines (26.4%) in the AL8/78-DGRH_1_ panel were identified using 35 SSR markers that contained at least one marker loss (hereafter called informative lines). The selected lines had deletion frequencies from 3–42.9% for the entire D-genome, and 20-100% for individual D-genome chromosomes. Among the 399 informative lines, 15-28% of the lines had deletions for an individual D-genome chromosome (Figure 3). As compared to 2.1% marker loss frequency of the entire DGRH_1_ panel, this selected panel of 399 lines had an average marker loss frequency of 9.9%.

#### ***Characterization of a subset of AL8/78-DGRH***_***1***_*** panel with larger set of markers and different marker systems***

**Figure 4:**
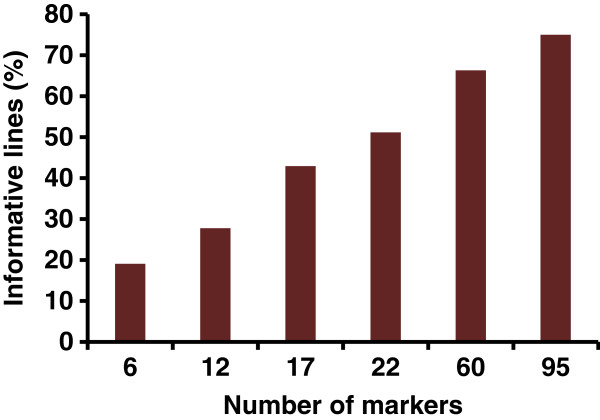
**Effect of number of markers on the detection of lines with deletions (informative lines).** The results are based on the characterization of 92 RH_1_ lines.

**Figure 5:**
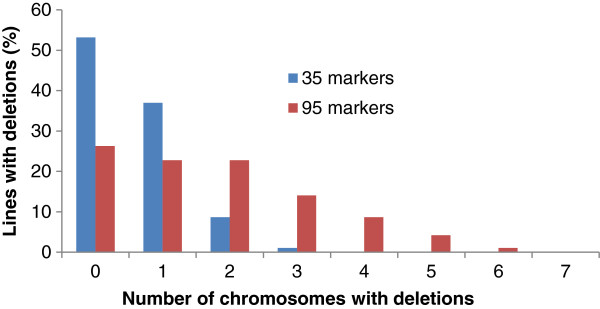
**Effect of number of markers in detecting RH lines with multiple chromosome breaks.** Percentage of RH_1_ lines with deletions for multiple chromosomes identified using two sets of different numbers of markers. The results are based on the screening of 92 lines.

The success of RH mapping depends on the deletions present in the panel. In order to have a deep insight into the deletion pattern in AL8/78-DGRH_1_ panel, and to study the effectiveness of a larger marker set for identifying lines with deletions, a set of 92 RH_1_ lines from the AL8/78-DGRH_1_ panel was also characterized with an additional 60 repeat DNA junction markers (RJMs) spanning the whole D-genome (total of 95 markers). Increasing the number of markers for characterizing the RH_1_ lines also increased the proportion of informative lines (Figure 4). In this set of lines, 35 SSR markers detected 46.7% lines with deletions, while 95 markers (35 SSR and 60 RJM) detected deletions in 73.9% of the lines. A drastic increase in the number of RH_1_ lines with multiple chromosome breaks (Figure 5) was also observed when characterizing the RH_1_ lines with 95 markers. With the 35 SSR markers, 79% of the lines with deletions showed marker loss for only a single chromosome, while the remaining 21% lines have deletions for two or three chromosomes. None of the lines showed deletions for four or more chromosomes. However, characterization of the same set of lines with 95 markers showed that 69% of the lines with deletions have marker loss for 2 to 6 chromosomes. While only 31% of the lines with deletions showed marker loss for a single chromosome (Figure 5). These results clearly suggest that a majority of the lines have more deletions which were not identified when using 35 markers.

Different type of DNA markers represent different distribution patterns across the chromosome and may lead to variability in the detection of deletions. In order to check the effectiveness of different marker systems in identification of deletions in the AL8/78-DGRH_1_ panel, the 92 RH_1_ lines were genotyped with RJM (eight), SSR (eight) and EST (fourteen) markers belonging to chromosome 2D. The SSR and EST markers represent nearly all of the deletion bins on chromosome 2D [42,43], while for RJMs, no such information was available and were picked randomly [44]. Average marker loss was significantly higher for RJMs (8.9%) compared to SSRs (3.8%) and ESTs (3.2%), which were not significantly different from each other. The RJMs also showed a much wider range of marker loss (1.1-19.5%), compared to SSRs (2.2-5.6%) or ESTs (1.1-5.6%). The frequency of marker loss was homogeneous for both SSR and EST markers (p<0.05), while it was significantly heterogeneous for RJMs (p<0.001).

**Figure 6:**
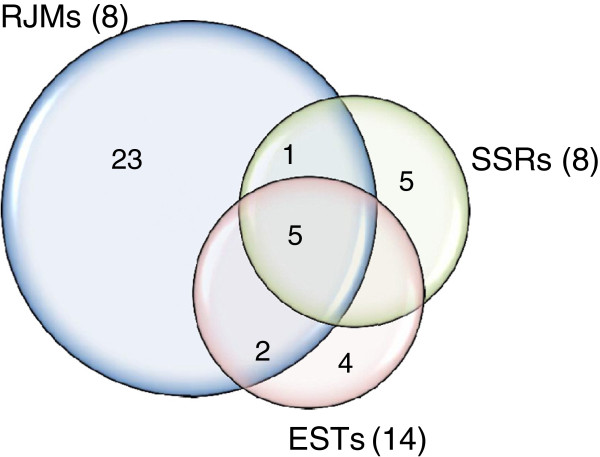
**Comparison of RJM, EST and SSR markers in detecting deletions.** Venn diagram shows the number of lines with deletions detected with each marker type in a set of 92 lines of the AL8/78-DGRH_1_ panel. Numbers of markers used from each type are in parenthesis.

The RJMs also detected almost three times (33.7%) more lines with deletions compared to SSR (12%) or EST (12%) markers. The numbers of lines with deletions detected by SSR and EST markers were the same. The data also shows that 80% of the lines have deletions for only one of the three marker systems while only 20% lines showed deletions for 2 or 3 marker types (Figure 6).

#### EST, SSR, RJM-based RH map for chromosome 2D

**Figure 7:**
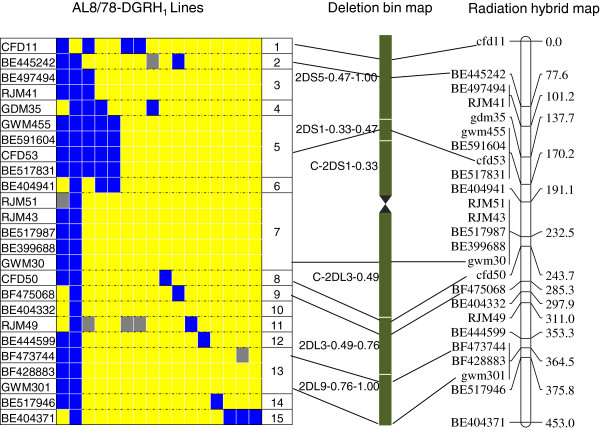
**A radiation hybrid map of chromosome 2D (right).** Also shown (left) is the graphical genotype for a panel of sixteen RH lines with deletions that dissected chromosome 2D into 15 different RH bins. Solid yellow squares indicate that the marker was present in the particular RH_1_ line. Solid blue squares indicate that the marker was absent in the particular RH_1_ line. Solid grey squares indicate missing information.

The genotyping (deletion typing) data for the 30 marker loci (14 ESTs, 8 SSR and 8 RJM) on 92 AL8/78-DGRH_1_ lines was used to construct a RH map for this chromosome. With the minimum LOD score of 3, a RH map based on 25 marker loci (14 ESTs, 7 SSR and 4 RJM) was constructed (Figure 7). These 25 markers were mapped to 15 unique positions and span a distance of 453 centi-Rays (cR) (Figure 7). A comparison of the 2D RH map with a published genetic map [38] shows a *cM/cR* ratio of 1:5.2. From the 92 RH_1_ lines genotyped, only 16 lines have deletions for the 25 markers with individual lines missing from one to 24 markers, for a total of 37 obligate breaks. This subset of 16 AL8/78-DGRH_1_ lines was able to dissect chromosome 2D into 15 distinct bins (Figure 7).

#### Resolution of AL8/78-DGRH_1_ panel

In order to check the mapping resolution of AL8/78-DGRH_1_ panel, the entire panel was analyzed with two RJM markers designed from the end sequences of two BAC clones belonging to a single contig. These RJM markers were mapped to the centromeric bin of 5DL and are located 400 kb apart (unpublished data). Four RH lines among the entire panel (1,510 lines) were identified as carrying a break between these two markers, suggesting a resolution of ~100 kb. The AL8/78-DGRH_1_ panel was further analyzed with three SSR markers physically mapped to the 1% of the most distal bin of 6DS (6DS6-0.99-1.00) which spans ~3.2 Mbp region [45]. Characterization of the entire RH_1_ panel with these SSRs identified 23 obligate breaks in this 3.2 Mbp region indicating a mapping resolution of <140 kb.

### Genetically effective cell number (GECN) in wheat

The GECN was calculated as explained by Hodgdon et al. [46] with some modifications as needed for radiations hybrids (Table 3, see material and methods for more details). In order to determine the GECN in the present study, 35 SSR marker (5 from each D-genome chromosome) based deletion data for 339 DGRH_1_ plants belonging to 29 RH_1_ families was used to calculate the segregation ratio for each unique deletion mutation. Deletion mutations for single chromosomes were treated separately. The number of progenies represented in an RH_1_ family ranged from 10 to 17. In total, 73 data sets were used to calculate the GECN. Based on the segregation ratio of deletions *vs.* wild type, the GECN in these families ranged from 1.4 to 8.5. However, the concept of GECN is more valid as an average [46,47] and the average GECN was calculated to be 5.25. The average GECN computed on the basis of deletions for individual chromosomes was not significantly different (Table 4). So, this data suggests that GECN in wheat is 5 or more. However, at higher GECN, a large number of progeny families are needed to identify all possible deletions.

**Table 3:** Genetically effective cell number (GECN)

**Segregation ratio**		**GECN**
**M**_**2 **_**population**	**RH**_**1 **_**population**	
3:1	1:1	1
7:1	3:1	2
11:1	5:1	3
15:1	7:1	4
19:1	9:1	5
23:1	11:1	6
27:1	13:1	7
31:1	15:1	8

**Table 4:** **Average GECN calculated from deletions on different chromosomes in the RH**_**1 **_**families**

**Chromosome**	**Average GECN**	**Range**	**RH**_**1 **_**families studied**
1D	5.2	1.8-8.5	21
2D	5.5	5.0-8.5	9
3D	5.5	1.7-8.5	7
4D	5.4	2.5-8.5	9
5D	4.7	1.4-8.5	9
6D	5.3	2.5-7.0	12
7D	5.4	2.8-7.0	6

## Discussion

Despite the fact that sequencing costs have reduced significantly in the past few years and may continue to do so in the future, short-read shotgun data without annotation does not provide the information needed for analysis of genetic and linkage data and its functional implications. Moreover, development of polymorphic markers and mapping of millions of contigs generated by next generation sequencing is prohibitively expensive despite the development of high-throughput genotyping platforms. Data from animal systems indicate that survey sequencing (1-2X genome coverage) combined with high resolution RH maps deliver a comprehensive map of over 10,000 loci in which over 90% of the markers were ordered [48]. When applied to plants, this combined approach can provide a dense physical map that may be used for crop improvement and selection, map-based gene cloning, and comparative analysis of various species. However, RH mapping relies heavily on the availability of an adequate RH population rich in detectable deletions. Wheat is an allohexaploid species, meaning that it contains three sets of homoeoloci for each gene and deletions in one or more of these loci can be potentially buffered by the others. Viable plants carrying large deletions can be obtained as demonstrated by the production of the wheat deletion bin genetic stocks [49]. The AL8/78-DGRH_1_ is a whole genome panel designed for the RH mapping of all seven D-genome chromosomes of *Ae. tauschii*.

### Radiation dose for development of RH panel

The selection of an appropriate dose for irradiation is an important step in the development of radiation hybrids in plants. Different genotypes may respond differently to radiation treatments. The hexaploid wheat cultivar Chinese Spring showed a survival rate of 21% when irradiated with 350 Gy of γ-rays [24] compared to 70% survival observed for the synthetic hexaploid wheat SW58 irradiated at the same dose. It was also observed that the seeds of LDN showed significantly higher survival compared to another durum wheat cultivar (both tetraploid) when irradiated with 350 Gy (unpublished data). Since SW58 contains the AB genome from LDN [40], a better response of SW58 and LDN to γ-radiation suggests that LDN genome may be more tolerant to radiation damage. Although the biological basis of irradiation tolerance in these lines is not known, the results of the present study suggests that for any RH panel, it is important to optimize the radiation dosage for the particular genotype.

The selection of an appropriate irradiation dose is also crucial as previous studies have observed an increased marker loss and mapping resolution with the increase in dose of γ-irradiation [18,22,24]. However, it is impossible with the RH approach in plants to go beyond a certain level of radiation as the increase in dosage leads to decrease in seed germination, plant survival, fertility and poor vigor [14,18,24]. A large decrease in plant survival above 350 Gy and lack of plant recovery from the 550 Gy irradiated seeds was observed in this study. This is likely due to the fact that the irradiated cells go through mitotic cell division and highly fragmented nuclei are not likely to survive the selection during plant development.

Based on the findings, the 450 Gy was the best possible dose for AL8/78-DGRH_1_ panel development. Additionally, as the higher doses of radiation cause higher number of breaks and rearrangements [18,22], a larger number of markers will be needed to develop linkage groups [9]. The 350 Gy RH lines were developed to complement the 450 Gy RH lines as the combination of RH panels of different resolution can yield a better contiguous map [50].

### Effect of crossing scheme and role of genome imprinting

The methodology presented here to develop a RH panel for the D-genome of *Ae. tauschii* involves two interspecific hybridizations. First, the synthetic hexaploid wheat SW58 (AABBDD) was developed by crossing tetraploid durum wheat LDN (AABB) to *A. tauschii* (DD) accession AL8/78 [40]. Secondly, the irradiated SW58 was crossed to LDN and pentaploid DGRH_1_ seeds were obtained, which allow for an exclusive identification of deletions in the D-genome of *Ae. tauschii*.

The F_1_ seeds developed from the interspecific crosses mostly show low germination and poor vigor that is attributed to possible crossing barriers. The most common post-zygotic reason for failure of wide hybridization is an embryo abortion due to poor endosperm development. The AL8/78-DGRH_1_ seeds developed from the reciprocal crosses (LDN × SW58 and SW58 × LDN) showed varying degree of endosperm development. The endosperm development was poor in most seeds when irradiated SW58 was used as male. The endosperm appeared more developed when SW58 was used as a female. This could be a reason for the differences observed in germination of AL8/78-DGRH_1_ seeds belonging to reciprocal crosses (~3% *vs.* ~80%). This difference could be attributed to genomic imprinting in endosperm resulting from differences in the ploidy levels of the two parental genotypes [51-53]. Endosperm is well developed whenever it is diploid [AAABBBDD in case of SW58 (♀) × LDN (♂)] for the whole set of extra seven chromosomes (DD in our case) and is wrinkled when it is haploid for the D chromosomes [AAABBBD in case of LDN (♀) × SW58(♂)] [54,55]. Although there are chances that homologous chromosomes of the D-genome have deletions, it is highly unlikely that both have the same deletion, therefore, endosperm containing a pair of D-genome chromosomes even with deletions has a better chance of development. The differences in endosperm may also be due to genomic imprinting which is the result of monoallelic gene expression in a parent-of-origin–dependent manner [56]. In Arabidopsis embryogenesis, there is a strong evidence of maternal epigenetic pathways controlling the parental contributions in plant embryos [57]. Additionally, in cases of interspecific crosses, the correct balance between maternal and paternal genomes is disturbed as in the cross between durum and synthetic hexaploid wheat, and crosses between species with different ploidy levels, leading to significant differences in the success of reciprocal crosses [53].

### GECN and its implication on RH panel development

When irradiating seeds, the diploid germline cells of the fully developed embryo are the radiation target. So, if the GECN is greater than one, the plant germinating from the M_1_ seed (RH_0_ seed in case of RHs) will be chimeric because independent mutations occur in each of the cells. The GECN has been reported to vary between different species. A GECN of two in *Arabidopsis thaliana*[58], *Nicotiana plumbaginifolia*[59-61], and soybean [62], four in flax [63], four or more in corn [47,64], and six in barley [46] have been reported. To our knowledge, this is the first time that the GECN is calculated from a RH_1_ population and for wheat. The information about GECN of the species under study is important in any RH or mutation-based experiments, as it can be used to estimate the rate of recovered deletions or other types of mutations. The probability of recovering a RH line with a deletion at a given locus in any RH family (or a mutant in M_2_ family) depends upon the number of plants screened from that family, the GECN for that particular plant species, and on the frequency of the mutation/deletion. The probability increases with the number of plants screened and decreases as the GECN increases. The AL8/78-DGRH_1_ panel represented 312 RH_1_ families, with an average of 4.8 siblings per family. With a GECN = 5 and screening of ~5 lines per RH_1_ family, approximately 40% of the deletions available in this panel for the 35 SSR marker loci were identified. The GECN of 5 indicates that at least 30 seeds in an RH_1_ family need to be analyzed to recover the same mutation at 95% confidence. Therefore, the GECN information for wheat is important in selecting informative lines from an RH_1_ population or identifying mutants in an M_2_ population. In wheat RH mapping, GECN of ≥5 offers an additional advantage of identifying multiple RH lines with different deletion patterns from the same RH_1_ family developed by a single cross. The 399 RH_1_ lines showing deletion(s) for one or more of the 35 SSR markers belonged to 211 crosses and showed up to five unique genotypes per cross. This indicates that more informative lines can be obtained from few crosses, thus, reducing the efforts required for developing a RH panel.

### Distribution of marker deletions in RHs

It has been estimated that less than 1% of recombination occurs in ~30% of the proximal regions of wheat chromosomes [5]. This means that map-based cloning of genes in the recombination-poor regions is not feasible utilizing the traditional recombination based approach. The uneven distribution of recombination also leads to variation in genetic to physical distances across the chromosome. RH mapping uses retention/loss frequency as a result of breaks caused by radiations, which are expected to be random. Therefore, the mapping power of RHs is expected to be uniform across the length of a given chromosome.

The five markers selected from each chromosome mostly represented different deletion bins and were genetically far from one another. The mapping data of these markers shows that the marker loss frequency within a chromosome is homogeneous for all chromosomes except for chromosome 1D and 7D. For 1D the heterogeneity was due to two markers showing higher retention. Similar results have also been reported earlier for the markers present in the same deletion bin (1DS5-0.70-1.00 and 1DL2-0.41-1.00) [14]. This could be due to the presence of critical gene(s) essential for the plant survival in this region leading to comparable lower observed marker loss than other chromosomal regions.

The distribution of deletions along a chromosome can be viewed from the five markers of chromosome 4D which represented different physical positions including centromeric bins of both long (C-4DL9-0.31) and short arm (C-4DS1-0.53) as well as sub-telomeric (4DS1-0.53-0.67) and telomeric region (4DL12-0.71-1.00). No preferential loss of markers for any region was observed. Similar results have been reported in maize [18], wheat [24], and cotton [22]. These results suggest that a uniform mapping resolution across the genome can be achieved with RHs, including the low recombination centromeric regions, which is not possible with genetic mapping. However, studies based on larger number of randomly distributed molecular markers of different types will provide a more detailed picture of the distribution of deletions along the length of the chromosome, and will help to determine, whether or not, the radiation-induced breakage is effected by chromosome structure or any other biological phenomenon.

### Selected RH lines for developing a high-resolution physical map of D-genome

The success of RH mapping depends on marker loss/retention frequency in a panel. Simulation studies have suggested that a retention/loss frequency of 50% would be optimal [65]. However, most RH mapping studies conducted in animals showed average retention frequency of about 20-30% [9]. The marker loss observed in this population was much lower than what was observed in animals, but was comparable to that earlier reported in wheat [24]. In plants, it is not possible to obtain the hybrids with marker loss comparable to human or animal RH panels as viable and fertile plants from the irradiated seeds need to be recovered.

The informative panel of 399 lines selected in this study has an average marker loss about five times higher (9.9%) than the whole unselected DGRH_1_ panel (2.1%). The effectiveness of this selection strategy was tested by screening a subset of 92 random RH_1_ lines with 60 additional markers. However, a high correlation (r=0.94) was observed between deletion frequency of the RH lines based on screening with a small (35) and large set (95) of markers. Moreover, almost all the lines (>92%) detected with multiple chromosome (>3) breaks using the larger set were also included in the set of informative lines identified using only 35 markers. This shows that the RH lines with deletions identified using few (35) markers have multiple deletions across the genome. Thus, the strategy to select highly informative lines using few markers from across the whole genome is as effective as selection based on almost three times larger marker set. However, selection with a larger set of markers detected additional informative lines, suggesting that the these lines possess smaller and unique deletions, which could be extremely useful when the purpose is to fine map a particular genomic region.

### Combination of different marker systems for a better contiguous map

Different marker systems represent different types of DNA sequences and potentially different distributions in the genome. Three different types of markers (RJM, SSR, EST) were used to characterize a subset of AL8/78-DGRH_1_ panel. The marker loss for RJMs was much higher and identified almost three times more lines with deletions as compared to SSRs or ESTs. A simple explanation would be that ESTs and SSRs mainly represent gene rich regions [66], potentially more prone to cause loss in vitality when deleted as compared to RJMs which usually span the inter-genic repetitive space [44]. Any deletion in the gene clusters will reduce the chances of survival of the particular RH line, leading to the detection of fewer lines with deletions among the surviving plants. Comparatively, plants with deletions in the repetitive DNA have more chances of survival. However, repetitive DNA is present in both genic and inter-genic regions [2,67] which could be a possible explanation for the wide range of marker loss (1.1-19.5%) observed for RJMs. The other explanation could be found in the specific marker design. RJMs are designed to span unique retrotransposon insertion sites. These insertion sites are typically characterized by a chromatin state more susceptible to DNA breakage [68]. Recent findings indicate that radiation damage and the subsequent DNA repair might also prefer these exposed chromatin regions to cause breakage [25]. This would also explain why RJMs have the tendency to be deleted with higher frequency. Similarly, the large amount of variation in chromatin state in intergenic regions would explain the wide range of marker loss for RJMs.

The abundance and uniform distribution of retrotransposons across the genome makes RJMs [44] ideal candidates for high density RH mapping. SSR markers, on the other hand, have been used extensively in wheat for the construction of genetic maps and the identification of marker-trait associations [69], http://wheat.pw.usda.gov. Therefore, SSRs can serve as excellent anchor points for mapping anonymous markers and as a linking bridge between the available genetic information and the high density RH maps. Gene-based markers like ESTs are an efficient tool for inter-species comparative genome analysis [15,38].

The use of different type of markers to characterize a subset of AL8/78-DGRH_1_ panel showed that deletions in the majority of the lines (80%) were detected by either of the three marker type. Similarly, a recent study in wheat reporting the first physical map of a wheat chromosome, which used different classes of marker types to anchor BAC contigs, observed that ~ 70% of the contigs were anchored with only one marker type [8]. These observations clearly suggest that different marker classes have different advantages for genomic research and also cover different regions of the genome. Therefore, a combination of various markers is better suited to obtain a complete RH/physical map for whole genome assembly.

### Combining RH and genetic mapping: A better approach for genome mapping

Dependence of genetic mapping on recombination makes it difficult to conduct map-based cloning of genes in the recombination-poor regions. The requirement of polymorphism for genetic mapping also makes it more difficult to map gene-based markers [12] which are invaluable tools for comparative studies across species. The selection pressure tends to remove any non-advantageous allelic polymorphisms in the gene space making gene-based markers highly monomorphic among individuals of the same species. In wheat, 9.9% polymorphism was observed for EST-derived SSRs compared to 35.5% polymorphism observed in case of genomic SSRs [12].

The presence/absence aspect of RH mapping which do not require genetic polymorphism may help in drastically increasing the number of mapped markers including gene-based markers. From the 14 ESTs mapped on the chromosome 2D using 92 random RH_1_ lines (only 16 were critical lines), only six could be mapped in a diploid bi-parental population of 572 F_2_ plants [38], suggesting that RH mapping has the potential to increase the number of mapped markers using comparatively fewer lines. The 2D-RH map also provided 5.2 fold higher resolution (*cM/cR* ratio of 1:5.2) than the genetic map [38], confirming that RH mapping indeed provides higher resolution than genetic mapping. At the same time, the availability of hundreds of genetic maps and genetically mapped loci in wheat [69] is an asset and this information may provide better consensus maps. In addition, the genetic maps could easily be used for identifying QTL/genes for any phenotypic trait segregating in that population. Therefore, RH mapping has the potential to complement a genetic mapping approach by mapping the markers in low recombination regions and making it possible to map a larger amount of monomorphic gene-based markers.

### Radiation hybrids for BAC contigs assembly

The IWGSC has established a clear road map to achieve the complete sequencing of the wheat genome [70]. This includes the creation of high quality physical maps for each of the 21 chromosomes, individually. In this direction, the first physical map of a single chromosome was reported for 3B [8]. The authors described the assembly of 1,036 contigs with an average size of 0.78 Mb, covering ~82% of the 3B chromosome. Although 1,443 molecular markers were developed from the BAC contigs, not all were polymorphic or could be uniquely mapped into a marker scaffold despite using information from 13 genetic mapping populations, especially for those regions that have limited recombination [8]. Apparently the use of genetic maps alone might not be enough to generate sufficient resolution for the IWGSC initiatives [8] due to non-uniform distribution of recombinants along the length of the chromosome and low level of polymorphism in bi-parental genetic populations. In humans [71,72] and animals [73-77], RH mapping played a major role in the contig assembly leading to whole genome sequencing [17].

Considering the success of RH approach in human and animal genome assembly, the AL8/78-DGRH_1_ panel was made to help the assembly of BAC contigs from the wheat D-genome project. Although an effort similar in scale to the one described here is not available as reference for the plant kingdom, preliminary studies suggested that a map resolution of ~200 kb is achievable with RH in plants [8,23,25]. Similar level of resolution was achieved in the AL8/78-DGRH_1_ panel as indicated by analysis with a set of markers for which the physical distance was known.

Past studies in plants have reported the detection of up to thirteen deletions for a single chromosome in an individual RH_1_ line [14,18,24,25]. However, as observed in the present study and also reported earlier [23] the number of detectable deletions may increase by genotyping the panel with more markers. Assuming an average of 3 random deletions for a particular chromosome and BAC contigs of a size observed by Paux et al. [8] for 3B (482 kb), the selected set of 399 highly informative AL8/78-DGRH_1_ lines is adequate to anchor and order all the BAC contigs of any D-genome chromosome.

The resources generated here provide a foundation for the development of next generation, high-density RH maps incorporating thousands of marker including SSRs, RJMs, ESTs etc. These maps will provide a marker scaffold to develop complete physical maps leading to the final sequence assembly of the seven wheat D-genome chromosomes.

### Potential applications of RHs for functional genomics

Cytogenetic stocks in wheat have been used extensively to study genes/phenotypes associated with a particular chromosome or its segment [78,79]. However, due to the availability of limited number of these lines, this approach offers an even lower resolution than genetic mapping. The association of phenotypic and genotypic data, both recorded on RH_1_ lines developed during the present study, may help assign the phenotype to genomic deletions as is done in case of genetic mapping or deletion bin mapping [78,79], but at much higher resolution. This has been successfully illustrated by mapping the genes in low recombination regions in wheat [14,80].

The first-generation radiation hybrids (RH_1s_) contain D-genome chromosomes with rearrangements in a monosomic condition. Variation is expected in the transmission of D-genome chromosomes to subsequent generations [81,82]. It also means that it will not be possible to replicate the phenotypic data recorded on the DGRH_1_ lines, unless the segregating lines are confirmed for the presence of a particular chromosome with the phenotypes. However, as an alternative RH maps can be used to identify the RH_1_ lines showing deletions for the markers associated with any particular trait of interest. Using this information, the parental M_2_ families of specific RH_1_ plants may be used to select homozygous and stable deletion mutants for the flanking markers (and the markers in between) of the gene/QTL. A set of such overlapping deletion lines and their trait data will help in physically placing the gene in a small segment of the chromosome. In a similar fashion, this approach may also be used for understanding the phenotypes associated with known genes (reverse genetics) by screening the DNA of the whole set of AL8/78-DGRH_1_ lines generated during the present study, for deletions of a particular gene sequence and then studying their parental M_2_s.

## Conclusions

Radiation hybrid mapping is an important tool for mapping/cloning genes in recombination poor regions [14] and developing a marker scaffold for whole genome assembly [8,25] of important crops like wheat with complex and highly repetitive genomes. A RH panel was developed for the D-genome of *Ae. tauschii* accession AL8/78 (AL8/78-DGRH_1_) and is the first reported for any wild ancestor of a cultivated plant species. Characterization of this panel with markers covering whole D-genome provided insight into various aspects of RH mapping which will be helpful in implementing similar tactics for important genomic studies in other plant species. Genetically effective cell number which has significant importance in developing RH panel was estimated for wheat for the first time. The results showed that the marker loss was independent of chromosome location and thus, more uniform resolution can be expected along the length of the chromosome including low recombination regions. Different marker systems mostly detected different RH lines with deletion, suggesting that a combination of marker systems is required to achieve a complete physical map. The results also showed that a much higher mapping resolution can be achieved with few informative RH lines compared to a larger recombinant population. Overall, the mapping resolution of this RH panel was estimated to be ~140 kb which is sufficient enough to align the available BAC contigs. The panel also showed a great potential for mapping gene based markers. Finally, a set of 399 most informative RH lines was also identified. This AL8/78-DGRH_1_ panel will be an invaluable resource for developing a complete marker scaffold for the whole genome sequence assembly as well as fine mapping and functional characterization of genes and gene networks present on the D-genome.

## Methods

### Seed material and radiation treatment

The present study used a synthetic hexaploid wheat line SW58 (*Triticum aestivum* L.; 2n=42; AABBDD) and a durum wheat cultivar Langdon (LDN; *Triticum durum* L*.*; 2n=28; AABB). SW58 was previously developed from a cross of LDN and *Ae. tauschii* (DD) accession AL8/78 [40]. Seeds from SW58 were equilibrated to 13% moisture as described earlier [14] and were γ-irradiated at five different doses {150, 250, 350, 450 and 550 Grays (Gy)}. The seeds were planted in the greenhouse after irradiation. Plant survival for each irradiation dose was determined as a proportion of surviving seedlings compared to the survival of plants from control seeds (non-irradiated SW58 seeds) one month after sowing.

### Population development

#### ***Crossing scheme***

After irradiation, the seeds (RH_0_) were pre-germinated in petri dishes. After one week of germination, the seedlings were transferred to six inch clay pots containing Sunshine Mix #1 (Sun Gro Horticulture, Vancouver, Canada) augmented with soil. Plants were grown in a greenhouse (16-h light cycle; 15°-25°C; water, pesticide and fertilizer applications as required). RH_0_ plants were crossed to normal LDN to obtain the D-genome RH_1_ (AL8/78-DGRH_1_) seeds (Figure 1). A 2,4-D hormone solution (213.05 mg/L of 2,4-D, 80 μl/L of Tween 80, 50mg/L of GA_3_, pH 10.36) was applied to the crossed spikes at 24 and 48 hours post pollination. After pollination, either the developing embryos were rescued from the growing RH_1_ seeds to obtain the RH_1_ plants, or the mature RH_1_ seeds were harvested and directly planted in the greenhouse to obtain the RH_1_ plants. Multiple RH_1_ seeds from the same cross were also planted to maximize the number of RH lines with different deletion events. All the RH_1_ plants from the same cross represent an RH_1_ family.

#### ***Embryo rescue***

The RH_1_ seeds were collected at 21–28 days after pollination. Seeds were surface-sterilized under a laminar flow hood by immersion in 70% (v/v) ethanol for 1 min., followed by 20% (v/v) household bleach for 5 min. The seeds were then rinsed three times for 5 min. each with sterilized water. Embryos were excised from the seeds under a dissecting microscope and placed on MS media [4.4 g/L of MS basal salt (Sigma-Aldrich, St. Louis, MO; M5519 with vitamins), 50 g/L of sucrose (Sigma-Aldrich, St. Louis, MO), 3.5 g/L of phyta gel (Sigma-Aldrich, St. Louis, MO), pH 5.7] in petri dishes. The petri dishes were sealed and kept at room temperature in the dark for embryo germination. After germination, embryos were kept under a 16-hours light cycle at room temperature. At the two leaf stage, the media was washed off the roots and the germinating seedlings were transplanted into a 96-well tray containing sunshine mix. The plants were transferred to soil beds after 15–20 days and grown under greenhouse conditions as described earlier.

### DNA markers for characterization of AL8/78-DGRH1 panel

Three different types of DNA marker were used in this study: simple sequence repeat (SSR), repeat DNA junction marker (RJM) and expressed sequence tag (EST). The SSR and EST markers were selected based on their known genetic or physical location [41-43]. For RJMs, only chromosome assignments were available and therefore, were picked randomly from different chromosomes [44]. The D-genome specificity of the markers was verified by positive amplification in hexaploid DNA (SW58; AABBDD) and no amplification in tetraploid wheat DNA (LDN; AABB). Since RH mapping is based on a presence/absence assay, the markers that amplified only a single D-genome specific fragment were multiplexed with an A or B genome-specific marker to provide a control against PCR failure. This internal multiplex control distinguished between absence of a marker band due to PCR failure (no bands present) and the deletion of the marker loci due to irradiation (only the control band present).

A total of 1,510 lines from the AL8/78-DGRH_1_ panel were genotyped with 35 SSR markers, 5 markers from each of the seven D-genome chromosomes [41,42]. Additionally, 92 RH_1_ lines (one 96 well plate with 4 parental genotypes for genotyping convenience) were further characterized with an additional 78 markers, which included 4 SSRs and 14 ESTs located on chromosome 2D and 60 RJMs representing all seven chromosomes of the D-genome [44].

To estimate the mapping resolution of the AL8/78-DGRH_1_ panel, two RJMs were developed from the end sequences of two BACs separated by *ca*. 400 Kb physical distance (unpublished data). Marker primers were designed using RJPrimers [83] and synthesized by Sigma-Aldrich (St. Louis, MO). The AL8/78-DGRH_1_ panel was then genotyped using these two RJMs to identify obligate breaks between them to provide an estimate of the physical resolution of the AL8/78-DGRH_1_ panel. Similarly, three SSR markers physically mapped to the 1% of the most distal bin of 6DS (6DS6-0.99-1.00) and covering a region of ~3.2 Mbp [45] were also used to identify obligate breaks and confirm the mapping resolution of the AL8/78-DGRH_1_ panel.

### DNA extraction and PCR analysis

DNA from parental genotypes and RH_1_ plants was extracted from lyophilized leaf tissue of one month-old plants as described by Guidet et al. [84]. PCR amplification was carried out in 15 μl reactions containing 1x PCR buffer, 1.5 mM of MgCl_2_, 200 nM of each dNTP, 250 nM of each primer, and 1.0 unit of *Taq* DNA polymerase. SSR and RJM markers were amplified in a thermocycler programed for initial denaturation at 94°C for 3 min.; 35–45 cycles of 94°C for 45 sec., 51°, 55°, 58° or 60°C (depending on annealing temperature) for 45 sec., and 72°C for 1 min.; followed by final extension at 72°C for 10 min. For EST markers a touchdown PCR was carried out with initial denaturation at 94°C for 5 min., 10 cycles of 94°C for 30 sec., 65°C for 30 sec. (reduced by 0.5°C each cycle) and 72°C for 1 min. followed by another 35 cycles of same conditions with annealing temperature of 60°C. A final extension was carried out at 72°C for 10 min. PCR products were separated on 3.0% w/v agarose gels in 0.5X TBE.

### Data analysis and RH map construction

The AL8/78-DGRH_1_ panel was scored for marker presence or absence. Ambiguous results in all cases were recorded as a missing data. Marker loss or retention frequency was defined as the proportion of RH lines with marker lost/retained in the AL8/78-DGRH_1_ panel. Chi-square tests were used to find differences in marker loss between radiation dosages, and to determine the homogeneity of marker loss between and within chromosomes.

A total of 30 markers (8 SSR, 8 RJM and 14 EST) belonging to chromosome 2D and screened on 92 lines of the AL8/78-DGRH_1_ panel, were used to construct a 2D RH map. The RH map was generated using Carthagene 1.2.2 [85]. Six SSR markers were first anchored based on available genetic information and deletion bin location [41,42]. Remaining markers with known bin information were then added to the anchored markers by mapping each bin separately. Every possible combination was attempted for each marker, and the lowest distance map was used as the framework map. The remaining markers were placed onto the framework map using the buildfw function through iterative analysis [25,86]. Two iterations were needed for this data set. Markers were assigned to intervals between two anchor markers using the command buildfw with LOD score of 3. These assigned markers, two initial inner and two outer anchor markers of the framework map were then mapped by Carthagene using build10, greedy search, genetic algorithm, annealing, flips, and polish functions. All markers mapped between two anchor markers underlying a specific interval were merged into the new framework map. All other markers were discarded and reused in the following iteration.

### Calculation of genetically effective cell number (GECN)

In a mature embryo, only a few meristematic cells give rise to the plant’s flowering parts. These cells (germ line) at the time of mutation induction which develop into an inflorescence structure, are called the genetically effective cells (GEC). The GECN was calculated using segregation ratios for deletions, according to the method of Li and Rédei [87] and Hodgdon et al. [46] with modifications as needed for the RH population (Table 3). In an M_2_ population, the progeny descended from a single (germ line) cell, is expected to show a simple Mendelian segregation ratio of 3:1 (AA, 2Aa, aa) for any recessive mutation at a given heterozygous locus (Aa in M_1_). However in case of RHs, deletion-type mutations are expected, so the RH_0_ plant is in a hemizygous condition (A-) for a deleted locus. Moreover, in the case of RHs, RH_0_ (Aa; same as M_1_) plants are not selfed, but rather are crossed to a second line which is null (−−) for the particular locus under study. Hence, RH_1_ plants originating from the same RH_0_ female plant (defined as RH_1_ family) segregate in a 1:1 (A-, --) ratio if the GECN =1. When the germline, at the time of the irradiation, comprises of more than one cell (GECN >1), an alteration of the segregation ratio will be observed (Table 3) since not all cells are expected to have identical deletions (or mutations) at a given locus in the same meristem at the same time.

Deletion data of 35 SSR markers for 339 DGRH_1_ plants belonging to 29 RH_1_ families was used to calculate the segregation ratio for each unique deletion mutation. Deletion mutations for individual chromosomes were treated separately. Using the frequency of a particular marker deletion mutation in RH_1_ families, the GECN in a RH_1_ population can be calculated as follows:

(1) GECN=t/2d

Where *t* is total number of siblings in a specific RH_1_ family, and *d* is the total number of siblings in a RH_1_ family with same deletion mutation.

## Abbreviations

BAC: Bacterial artificial chromosome; cR: Centi-Rays; GECN: Genetically effective cell number; Gy: Gray; LDN: Langdon; IWGSC: International wheat genome sequencing consortium; RH: Radiation hybrid; RJM: Repeat DNA junction marker.

## Competing interests

The authors declare that they have no competing interests.

## Authors’ contributions

AK and SFK designed research; AK, KS and MMdeJ performed the research; AK, KS, MJI, FB, FG and PMAK analyzed the data; TD, YW, MCL, YQG, GRL and JD provided markers; SSX provided seed material; OAlA and AMD developed iterative framework mapping script; AK drafted the manuscript with comments from all authors; MJI, FG and SFK managed the project. All authors read and approved the final manuscript.
